# Bacterial N_2_O mitigation potential in soil-based systems and liquid cultures: a comprehensive meta-analysis

**DOI:** 10.3389/fmicb.2026.1803828

**Published:** 2026-04-08

**Authors:** Shengsen Zhou, Ruixuan Zhu, Yongfeng Sun, Dingjiao Peng, Fumin Wei, Xiaomai Yuan, Beilei Wei, Dan Lu, Weiwei Li, Yu Jiang, Ziting Wang

**Affiliations:** 1 State Key Lab for Conservation and Utilization of Subtropical Agri-Biological Resources, Guangxi Key Lab for Sugarcane Biology, College of Agriculture, Guangxi University, Nanning, China; 2 College of Agriculture, Guangxi University, Nanning, China; 3 Nanjing Agricultural University, Nanjing, China

**Keywords:** environmental systems, meta-analysis, microbial mitigation, nitrous oxide, NosZ gene

## Abstract

The biological mitigation of nitrous oxide (N_2_O), a significant greenhouse gas, plays a crucial role in slowing global climate change. Using a meta-analysis approach, this study systematically integrated 257 data points from 34 independent studies to quantitatively assess the regulatory effects of bacterial inoculation on N_2_O mitigation across diverse agricultural environments. Our analysis revealed that environmental system emerged as the most critical factor determining mitigation effectiveness, leading us to focus on two contrasting incubation systems: soil-based and liquid culture environments, for which potential molecular mechanisms were explored through functional gene analysis and phylogenetic characterization. Results demonstrated that bacterial inoculation significantly reduced N_2_O cumulative emissions and enhanced reduction rates, with effects exhibiting pronounced environment-specificity: liquid culture systems achieved a mitigation extent of 68.3%, superior to the 42.9% observed in soil-based systems, with corresponding N_2_O reduction rates of 3.68 and 1.78 μmol·h^−1^·g^−1^, respectively. Environmental factor analysis revealed that bacterial inoculation in soil-based systems showed lower net N_2_O accumulation under microoxic conditions, with greater effectiveness in acidic environments and during medium-term cultivation; whereas liquid culture systems achieved rapid mitigation response under anoxic neutral conditions. Functional gene analysis revealed potential synergistic regulatory patterns of multiple metabolic pathways including denitrification, dissimilatory nitrate reduction, and nitrogen fixation, with the denitrification pathway likely playing a core mitigation role in both environment types. This study revealed the environment-specificity of N_2_O biological mitigation and identified *Bradyrhizobium* and *Azospira* as key functional genera in soil-based and liquid culture systems, respectively, providing scientific guidance for developing environment-specific microbial mitigation technologies.

## 1 Introduction

Nitrous oxide (N_2_O), as the third most important greenhouse gas, possesses an atmospheric lifetime of approximately 116 years (Prather et al., 2015) and a global warming potential approximately 265 times greater than CO_2_, and serves as a primary contributor to stratospheric ozone depletion (Ravishankara et al., 2009). Agricultural activities constitute the predominant source of global N_2_O emissions (>70%): driven by global population growth and agricultural intensification, the increased application of nitrogen fertilizers in agriculture has led to a significant rise in N_2_O emissions, with global N_2_O emissions projected to increase by 35%−60% by 2050, posing severe challenges to achieving the emission reduction targets of the Paris Agreement (Bahram et al., 2022; Thompson et al., 2019). Therefore, there is an urgent need to develop effective mitigation strategies for agricultural N_2_O emissions to control global greenhouse gas emissions.

Microorganisms play a pivotal regulatory role in the global nitrogen cycle, wherein those that harbor the *nosZ* gene catalyze the conversion of N_2_O to N_2_ through the encoded N_2_O reductase, a process that represents the only known biological N_2_O consumption pathway (Sanford et al., 2012). The *nosZ* gene encompasses two major evolutionary clades (clade I and clade II), with clade I *nosZ* predominantly present in conventional denitrifying bacteria, while clade II *nosZ* is commonly found in non-denitrifying bacteria and demonstrates enhanced N_2_O reduction activity in environments with low N_2_O concentrations, thus theoretically conferring superior mitigation potential (Yoon et al., 2016). These attributes provide crucial theoretical foundations for the screening of N_2_O mitigation strains. Concurrently, with the continuous discovery of novel mitigation strains such as *Stutzerimonas stutzeri* and *Cloacibacterium* sp., the strain resource pool for N_2_O biological mitigation is becoming increasingly enriched (Hiis et al., 2024; Bano et al., 2024). This expanding strain diversity provides enhanced options for crafting precise mitigation strategies while highlighting the necessity for systematic evaluation of functional characteristics across different strains.

However, the functional expression of microbial N_2_O mitigation capacity exhibits pronounced context dependency, governed by multifaceted interactions among physicochemical parameters and environmental heterogeneity. The catalytic efficiency of N_2_O reductase, encoded by the nosZ gene, demonstrates high sensitivity to environmental modulators including pH, redox conditions, and metal cofactor availability (Hallin et al., 2018). The spatiotemporal heterogeneity of environmental conditions (such as variations in physical architecture, moisture regimes, and oxygen gradients) fundamentally shapes the distribution and kinetics of N_2_O reduction processes. This heterogeneity drives divergent metabolic responses in microbial communities across distinct biogeochemical microenvironments (Graf et al., 2014). Despite accumulating evidence from individual studies, these results are often difficult to compare across environments due to diverse experimental protocols, and single-condition findings may not adequately represent bacterial performance across agricultural systems. This fragmentation has prevented the identification of universal mitigation mechanisms versus environment-specific responses, hindering the development of broadly applicable strategies (O'Callaghan et al., 2022). Meta-analysis addresses these limitations by systematically integrating effect sizes across multiple studies while accounting for study-specific heterogeneity, enabling extraction of robust patterns that reveal both conserved mechanisms and context-dependent responses (Gurevitch et al., 2018). This approach is particularly powerful for comprehensive evaluation of microbial N_2_O mitigation across different environmental systems, providing essential insights for formulating environment-based differentiated agricultural mitigation strategies.

Based on the aforementioned background, this study employed a meta-analysis approach to systematically integrate 257 data points from 34 independent studies across diverse environmental systems. The research objectives were: (1) to quantify the regulatory effects of bacterial inoculation on N_2_O mitigation; (2) to identify the influence patterns of key physicochemical factors across different environmental systems; (3) to evaluate the molecular regulatory mechanisms of 30 nitrogen cycle-related functional genes and 13 metabolic pathways on N_2_O mitigation; and (4) to systematically screen dominant mitigation genera with superior performance characteristics. Through these analyses, this study aimed to elucidate the environment-specific mechanisms of microbial N_2_O mitigation processes and provide a theoretical basis for formulating differentiated microbial mitigation strategies tailored to different environmental systems.

## 2 Materials and methods

### 2.1 Literature search and selection

This study conducted a systematic and comprehensive search of peer-reviewed literature up to April 2025 using the following databases: Web of Science (https://www.webofscience.com/wos/), Scopus (https://www.scopus.com/), China National Knowledge Infrastructure/CNKI (https://www.cnki.net/), Wanfang Data Knowledge Service Platform (https://www.wanfangdata.com.cn/), and Google Scholar (https://scholar.google.com/). The following search terms were used: “nitrous oxide OR N_2_O,” “sink OR consumption OR reduction OR uptake OR negative flux,” “denitrification OR *nosZ* OR N_2_O reductase,” and “bacteri^*^.”

Inclusion criteria were: (1) studies reporting data on N_2_O emissions or reduction rates with bacterial inoculation treatment, where axenic bacterial cultures (pure cultures of single strains) were grown under laboratory conditions and then inoculated into experimental systems; studies using enrichment cultures, mixed microbial consortia, or non-inoculated treatments (relying solely on native soil microbiota) were excluded; (2) clear experimental conditions and bacterial taxonomic information; (3) research conducted in diverse environmental systems, including soil-based systems (various soil types, soil extracts, soil suspensions) and liquid culture systems (liquid media simulating soil chemical conditions). A total of 257 data points from 34 independent studies were ultimately included in the meta-analysis. The complete literature screening process is detailed in the PRISMA flow diagram (Supplementary Figure S1). A complete list of all included studies is provided in Supplementary Text S1, which includes both English and Chinese literature sources.

### 2.2 Data extraction and functional gene characterization

#### 2.2.1 Basic data collection

For different data presentation formats in the literature, tabular and textual data were directly extracted, while graphical data were digitized using GetData Graph Digitizer (V2.5; S. Fedorov, Russia) software. The collected information included: (1) study metadata (authors, publication year); (2) microbial information (genus, species); (3) N_2_O emission parameters (cumulative N_2_O emissions, expressed as mg N_2_O-N kg^−1^ soil; N_2_O reduction rates, expressed as μmol·h^−1^·g^−1^). For values not directly reported in source studies, cumulative N_2_O emissions were calculated as Σ(Flux_i_ × Δt_i_), where Flux_i_ is the emission flux at time point *i* and Δt_i_ is the time interval; The N_2_O reduction rate was calculated as follows: N_2_O reduction rate = (N_2_O consumed)/(*M* × *t*), where *M* is the total bacterial dry weight biomass (g) and *t* is the incubation time (h), yielding reduction rates in units of μmol·h^−1^·g^−1^. When biomass was reported in alternative units (protein content or cell counts), we standardized measurements to dry weight equivalents using species-specific conversion factors when available from the original studies, or established conversion approaches (Bratbak and Dundas, 1984; Fagerbakke et al., 1996; Roller et al., 2016). This normalization enabled direct comparison of N_2_O reduction rates across studies with different bacterial strains and experimental conditions; (4) environmental system type, physicochemical factors, and cultivation time as categorized in Section 2.4.2; (5) functional gene and metabolic pathway data; and (6) microbial 16S rRNA gene sequence information for phylogenetic analysis. Data used in this meta-analysis are provided in the Supplementary Data File.

#### 2.2.2 16S rRNA gene sequence acquisition and processing

16S rRNA gene sequence information of bacterial strains was extracted from the included studies. When sequence data were directly provided in the literature, the sequences were obtained directly; when studies only provided strain names, the corresponding 16S rRNA gene sequences were retrieved from the NCBI GenBank database. All sequence data underwent quality control, with sequences shorter than 1,200 bp removed, thereby retaining high-quality full-length or near-full-length 16S rRNA gene sequences.

#### 2.2.3 Functional gene and metabolic pathway analysis

For each bacterial genus identified in the included studies, representative genome sequences were retrieved from the NCBI GenBank database, prioritizing complete or high-quality draft genomes. Functional gene identification was conducted using BLAST+ (version 2.12.0; National Center for Biotechnology Information, Bethesda, MD, United States) (Camacho et al., 2009) with query sequences from the FunGene database (Fish et al., 2013), targeting nitrogen cycle-related genes across denitrification, nitrification, nitrogen fixation, dissimilatory nitrate reduction, and anaerobic ammonium oxidation pathways (Chen et al., 2024; Thakur and Gauba, 2023). Detection criteria (*E*-value < 1e-5, sequence identity > 60%, query coverage > 50%) were established following validated protocols for nitrogen cycle gene annotation (Orellana et al., 2014; Jones et al., 2013), which balance the detection of functionally relevant sequence variants while minimizing false positive annotations. Identified genes were mapped to KEGG nitrogen metabolism pathways using the KEGG Mapper tool (Kanehisa and Sato, 2020) to assess pathway completeness in each bacterial genus. Gene presence was recorded as binary data (1 = present, 0 = absent) for each strain based on genome annotation results. The genomic annotation data were integrated with N_2_O mitigation effect sizes through subgroup meta-analysis.

### 2.3 Phylogenetic analysis

Using maximum likelihood, we constructed a phylogenetic tree based on 16S rRNA gene sequences from 84 nitrogen cycle-related bacterial strains obtained from the 34 studies in this meta-analysis. The 16S rRNA gene sequences were retrieved from NCBI GenBank using accession numbers provided in the literature. Sequence alignment was carried out using the MUSCLE algorithm, followed by phylogenetic reconstruction using IQ-TREE software, with evolutionary distances displayed at a scale of 0.1 substitutions per site. Bootstrap support values were assessed through 1,000 bootstrap resampling analyses, with support values >70% deemed reliable phylogenetic relationships.

### 2.4 Statistical analysis

Meta-analysis was performed using a random-effects model, with analyses conducted using R software (v4.4.2; R Foundation for Statistical Computing, Vienna, Austria) and the “metafor” package.

#### 2.4.1 Effect size analysis

In this meta-analysis, the natural logarithmic response ratio (*RR*) of variables under treatment and control conditions was calculated to indicate the magnitude of response to bacterial inoculation. The *RR* and variance were computed using the formulas shown in Equations 1 and 2 (Hedges et al., 1999):


$$\begin{array}{l} \text{Effect size}=\ln\;RR=\ln\left(X_{t}/X_{c}\right) \end{array}$$ (1)

where *X*_*t*_ and *X*_*c*_ represent the mean values of the bacterial treatment and control groups, respectively. For N_2_O cumulative emissions, ln*RR* < 0 indicates mitigation effects; for N_2_O reduction rates, ln*RR* > 0 indicates promotion effects.

The variance (*V*) of effect size was calculated as follows:


$$\begin{array}{l} V=\frac{S_{t}^{2}}{N_{t}X_{t}^{2}}+\frac{S_{c}^{2}}{N_{c}X_{c}^{2}} \end{array}$$ (2)

where *S*_*t*_ and *S*_*c*_ represent the standard deviations of the treatment and control groups, respectively, and *N*_*t*_ and *N*_*c*_ represent the number of replicates in the treatment and control groups, respectively.

A random-effects model was employed to evaluate the effects of bacterial inoculation on different variables. The weighting coefficient (*w*) and weighted mean ln*RR*_++_ were computed as shown in Equations 3 and 4:


$$\begin{array}{l} Wi=1/vi \end{array}$$ (3)

$$\begin{array}{l} \ln RR_{++}=\frac{\sum_{i=1}^{k}\left(wi\times \ln\;RRi\right)}{\sum_{i=1}^{k}wi} \end{array}$$ (4)

where *v*_*i*_ represents the variance. The weighted log response ratio and 95% confidence intervals (CI) were calculated. To facilitate intuitive visualization and interpretation of the meta-analysis results, the weighted response ratio was converted into percentages as shown in Equation 5:


$$\begin{array}{l} \text{Change}\left(\%\right)=\left(\exp\left(\ln RR_{++}\right)-1\right)\times 100 \end{array}$$ (5)

#### 2.4.2 Subgroup analysis

Subgroup analyses were conducted to evaluate the moderating effects of different factors on bacterial N_2_O mitigation. Factors were classified into four categories based on their ecological and experimental characteristics:

(1) Environmental system: this classification distinguished between the fundamental experimental contexts in which bacterial mitigation was evaluated. Soil-based systems included experiments conducted in natural soil, soil extracts, or soil suspensions that retained the physical structure and microbial community characteristics of soil environments. Liquid culture systems included experiments performed in liquid media that simulated specific soil chemical conditions but lacked soil's physical matrix and indigenous microbial communities. (2) Phylogenetic classification: bacterial isolates were grouped at the genus level based on their taxonomic affiliations determined from 16S rRNA gene sequences. This classification allowed evaluation of whether phylogenetically related bacteria exhibited similar N_2_O mitigation patterns. (3) Physicochemical factors: this category encompassed chemical and physical parameters that varied within experimental systems. pH regimes were classified as acidic (pH < 6.5), neutral (pH 6.5–7.5), or alkaline (pH > 7.5) based on the initial values reported in the original studies, representing the environmental conditions at the time of bacterial inoculation. Oxygen status was classified according to oxygen availability during incubation. Based on microbial physiological adaptability and metabolic characteristics under different oxygen gradients (Prakash et al., 2025; Morris and Schmidt, 2013), anoxic conditions were defined as oxygen concentrations below 1%, microoxic conditions as 1%−10% O_2_, and aerobic conditions as >10% O_2_. These oxygen classifications reflected experimental cultivation conditions rather than *in situ* soil oxygen status. Soil nutrient properties included total carbon (TC), nitrate (${\text{NO}}_{3}^{-}$), ammonium (${\text{NH}}_{4}^{+}$), available phosphorus (AP), and available potassium (AK), with concentration ranges categorized based on data distribution to evaluate dose-dependent effects. Subgroup analyses of these soil nutrient parameters were conducted only for soil-based systems, as liquid culture systems employ standardized artificial media with controlled and relatively uniform nutrient compositions; accordingly, analyses for liquid cultures focused on the experimentally manipulated factors of pH, oxygen status, and cultivation time. (4) Cultivation time subgroups: Soil-based systems were divided into < 10, 10–60, and >60 days; liquid culture systems were divided into < 1, 1–5, and >5 days. These distinct temporal groupings reflect the different microbial growth rates and response timescales between soil-based and liquid culture systems. Groupings were determined based on data distribution patterns within each system type, referencing the staggered change patterns of microbial communities during post-disturbance recovery processes (Ferrenberg et al., 2013; Jurburg et al., 2017) and the response time of soil microbial communities to environmental changes (Sezonov et al., 2007).

#### 2.4.3 Publication bias assessment

Publication bias for all collected parameters was assessed using Rosenthal's fail-safe number method. The fail-safe number indicates how many additional studies with null results would be needed to reduce the overall effect size to non-significance. When Rosenthal's fail-safe number exceeds the threshold of 5*n* + 10 (where *n* is the number of observations), it indicates no significant publication bias in the meta-analysis (Li et al., 2024). Results showed that only cumulative N_2_O emissions (*n* = 212, *p* < 0.0001) and N_2_O reduction rate (*n* = 214, *p* < 0.0001) demonstrated statistically significant effects with fail-safe numbers (6,655 and 15,973, respectively) substantially exceeding their thresholds (1,070 and 1,080, respectively). Other parameters, including ${\text{NH}}_{4}^{+}$, ${\text{NO}}_{3}^{-}$, PNR, PDR, and qPCR data for functional genes (*nirS, nirK, nosZ*), either showed non-significant effects (*p* > 0.05) or had insufficient data availability across studies. Therefore, cumulative N_2_O emissions and N_2_O reduction rate were selected as the primary outcome variables for subsequent meta-analysis (Supplementary Table S1).

#### 2.4.4 Heterogeneity test

Prior to conducting subgroup analyses, the QM (*Q*-test for moderators) statistic was employed to test for heterogeneity of effects of various environmental factors on cumulative N_2_O emissions and N_2_O reduction rates, identifying key variables with significant regulatory effects.

#### 2.4.5 Statistical significance assessment

Differences between subgroups were tested using the *Q* statistic, with *p* < 0.05 considered statistically significant. The significance level for all statistical analyses was set at α = 0.05. In reporting statistical results, significance was expressed as follows: *p* < 0.001 (extremely significant, ^***^), *p* < 0.01 (highly significant, ^**^), *p* < 0.05 (significant, ^*^), and *p* ≥ 0.05 considered non-significant.

## 3 Results

### 3.1 Effects of bacterial inoculation on N_2_O emissions and reduction rates

**Figure 1:**
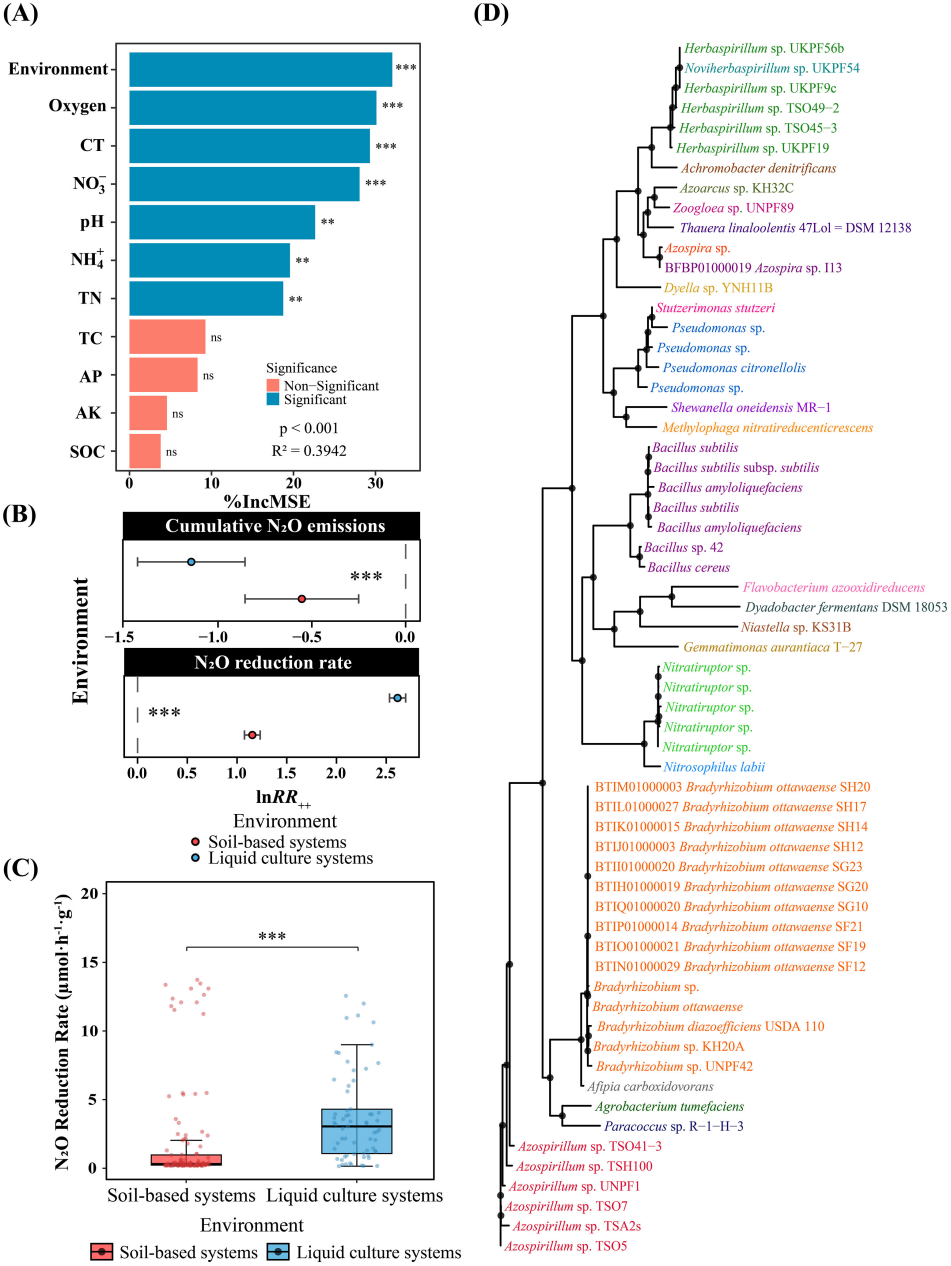
Effects of environmental system and physicochemical factors on bacterial N_2_O mitigation. **(A)** Random forest variable importance analysis (%IncMSE; *R*^2^ = 0.3942). Environment: soil-based vs. liquid culture systems. Physicochemical factors: pH, oxygen status, cultivation time (CT), TN, TC, ${\text{NH}}_{4}^{+}$, ${\text{NO}}_{3}^{-}$, AP, AK, and SOC. **(B)** Effects of bacterial inoculation on cumulative N_2_O emissions (lnRR++). **(C)** N_2_O reduction rates in both systems. **(D)** Phylogenetic tree based on 16S rRNA gene sequences from 84 strains. Red: soil-based systems; blue: liquid cultures. Error bars: 95% CI. ***p < 0.001, **p < 0.01, *p < 0.05.

Random forest analysis identified environmental system as the most critical factor determining N_2_O mitigation effectiveness, with high variable importance (%IncMSE = 31.8) and a significant contribution to model performance (*R*^2^ = 0.3942, Figure 1A). This analysis justified focusing subsequent analyses on environmental comparisons and revealed that bacterial inoculation had significant positive regulatory effects on N_2_O metabolism, with pronounced environment-specificity (Figures 1B, C). Bacterial inoculation achieved a 68.3% N_2_O mitigation effect in liquid culture systems (ln*RR*_++_ = −1.15), which was significantly superior to the 42.9% mitigation effect in soil-based systems (ln*RR*_++_ = −0.56). This environment-specific effect was even more pronounced for N_2_O reduction rates: the mean N_2_O reduction rate in liquid culture systems was 3.68 μmol·h^−1^·g^−1^, significantly higher than the 1.78 μmol·h^−1^·g^−1^ in soil-based systems. Phylogenetic analysis revealed that genera with the capacity to mitigate N_2_O exhibited multiple evolutionary origins, including Proteobacteria, Firmicutes, and Actinobacteria, reflecting the combined effects of horizontal gene transfer and environmental selection pressure (Figure 1D).

### 3.2 System-specific effects of physicochemical factors on N_2_O mitigation

**Figure 2:**
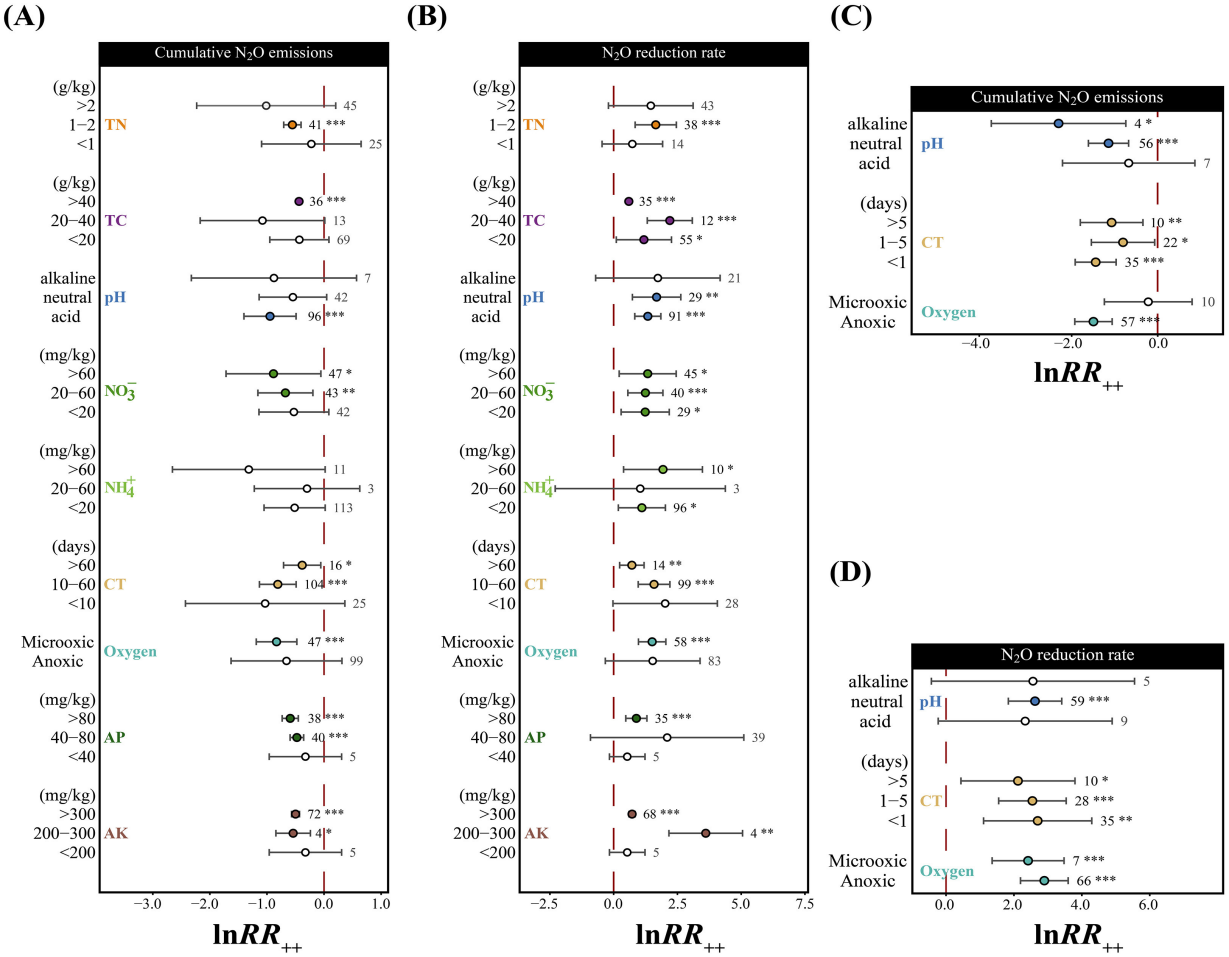
Effects of physicochemical factors on cumulative N_2_O emissions and N_2_O reduction rates in soil-based systems and liquid culture systems. Soil-based systems: **(A)** Cumulative N_2_O emissions; **(B)** N_2_O reduction rates; Liquid culture systems: **(C)** Cumulative N_2_O emissions; **(D)** N_2_O reduction rates. Physicochemical factor abbreviations are as defined in Figure 1A. Error bars represent 95% confidence intervals, numbers indicate study counts. Significance levels: ***p < 0.001, **p < 0.01, *p < 0.05, unmarked indicates non-significant. Red dashed line: zero effect line, effect size represents log response ratio.

The regulation of N_2_O mitigation effects by physicochemical factors exhibited significant system-specific patterns (Figure 2). Heterogeneity tests revealed significant statistical heterogeneity in N_2_O mitigation effects between different environmental systems (Supplementary Table S2), suggesting that physicochemical factors may exhibit distinct regulatory patterns across soil-based and liquid culture systems. In soil-based systems, N_2_O mitigation effects under microoxic conditions were significantly superior to those under anoxic conditions (*p* < 0.001), acidic conditions (pH < 6.5) demonstrated the strongest mitigation effects (*p* < 0.001), and medium-term cultivation (10–60 days) showed the most significant mitigation effects (*p* < 0.001; Figure 2A). Among soil physicochemical properties, total carbon (TC) and nitrate (${\text{NO}}_{3}^{-}$) both exhibited significant regulatory effects on N_2_O reduction rates (*p* < 0.05 to *p* < 0.001). TC showed significant effects on N_2_O cumulative emissions at high concentration ranges (>40 g/kg), while ${\text{NO}}_{3}^{-}$ exhibited significant regulatory effects on both cumulative emissions and reduction rates at moderate to high concentration ranges (>20 mg/kg). Ammonium (${\text{NH}}_{4}^{+}$) showed no significant effects on cumulative emissions, but significantly influenced N_2_O reduction rates within specific concentration ranges. Additionally, high concentrations of available phosphorus (AP; >80 mg/kg) and available potassium (AK; >300 mg/kg) both significantly influenced N_2_O cumulative emissions and reduction rates (Figures 2A, B). In liquid culture systems, environmental regulatory factors were relatively simplified, with anoxic conditions exhibiting the strongest effects on both N_2_O cumulative emissions and reduction rates (*p* < 0.001), pH showing optimal effects under neutral conditions (*p* < 0.01), and short-term cultivation (< 1 day) displaying the strongest rapid response effects (*p* < 0.001; Figures 2C, D).

### 3.3 Environment-specific performance of dominant genera

**Figure 3:**
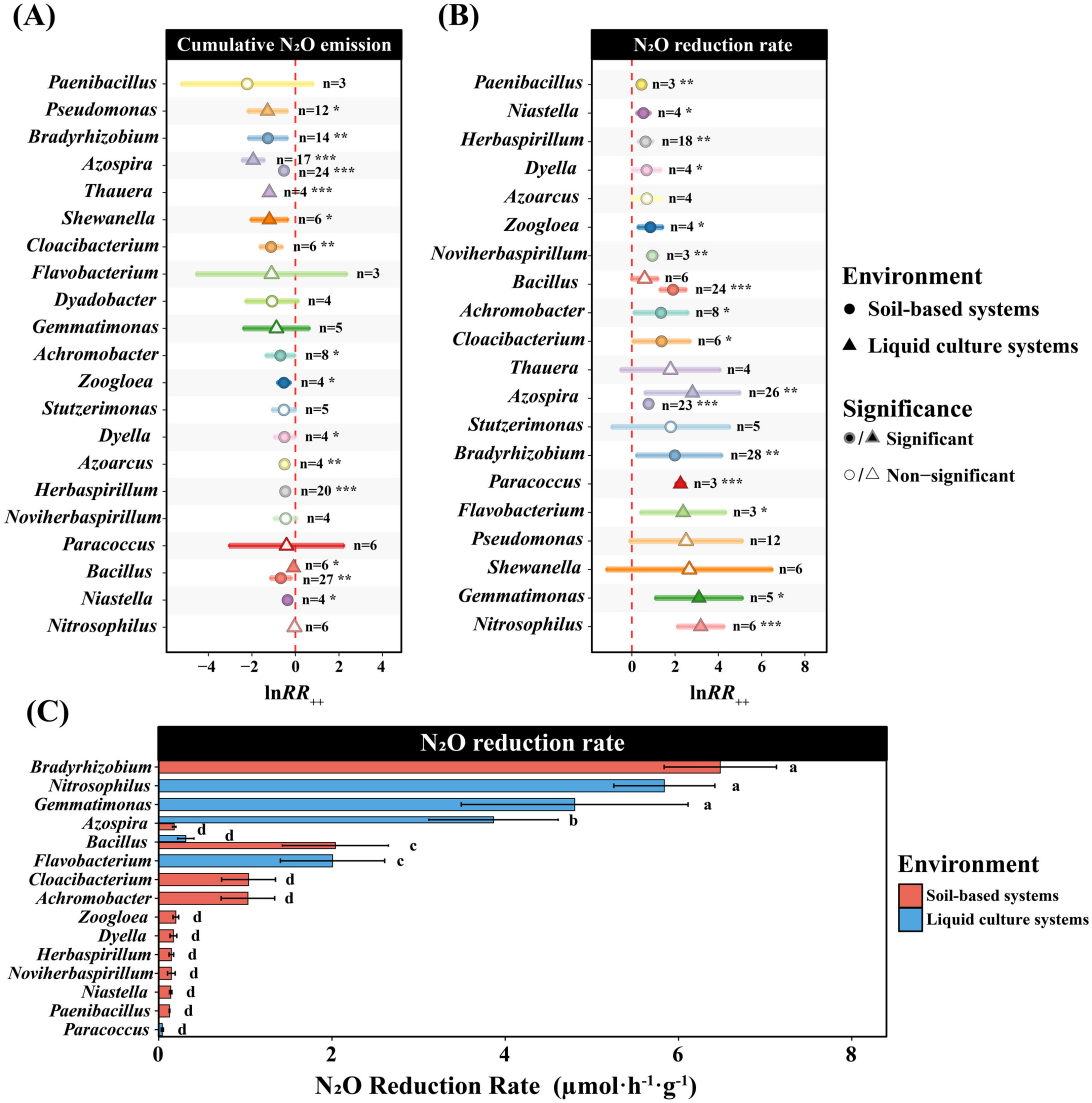
Differential effects of bacterial genera on N_2_O mitigation across soil-based and liquid culture systems. **(A)** Forest plot showing weighted log response ratios (lnRR_++_) for cumulative N_2_O emissions across bacterial genera. **(B)** Forest plot showing weighted log response ratios (lnRR_++_) for N_2_O reduction rates across bacterial genera. In panels **(A)** and **(B)**, each data point represents the meta-analytic effect size for a genus in a specific environment. Circles and triangles indicate soil-based and liquid culture systems, respectively. Filled symbols denote significant effects (*p* < 0.05), while open symbols indicate non-significant effects. Significance levels: ***p < 0.001, **p < 0.01, *p < 0.05. **(C)** Comparison of actual N_2_O reduction rates (μmol h^−1^) among genera that showed significant effects in at least one environment in panel **(B)**. For each selected genus, actual reduction rates are displayed for environments where sufficient data were available (*n* ≥ 3 individual observations per genus-environment combination). Bars represent mean values of individual observations. Error bars indicate standard error. Different lowercase letters indicate significant differences within each environment (*p* < 0.05).

Meta-analysis revealed significant differences in N_2_O mitigation efficacy among bacterial genera, with performance patterns varying between soil-based and liquid culture systems (Figure 3). Regarding cumulative N_2_O emissions, *Bradyrhizobium* showed significant mitigation effects in soil-based systems (ln*RR*_++_ = −1.24, 95% CI: −2.11 to −0.41, *p* < 0.001), while *Azospira* exhibited a mitigation effect value of approximately −1.9 in liquid culture systems (95% CI: −2.41 to −1.38, *p* < 0.001). For N_2_O reduction rates, *Nitrosophilus* showed the highest effect value (ln*RR*_++_ = 3.15, 95% CI: 2.12–4.25, *p* < 0.05), followed by *Gemmatimonas* (ln*RR*_++_ = 3.05, CI: 1.12–5.19, *p* < 0.001). Quantitative analysis revealed that *Bradyrhizobium* achieved the highest N_2_O reduction rate in soil-based systems (6.48 μmol·h^−1^·g^−1^), while *Azospira* showed a reduction rate of 3.96 μmol·h^−1^·g^−1^ in liquid culture systems. Multiple comparison analysis indicated significant differences in reduction rates among different genera (*p* < 0.05, Figure 3C).

### 3.4 Regulatory effects of nitrogen cycle functional genes and metabolic pathways

**Figure 4:**
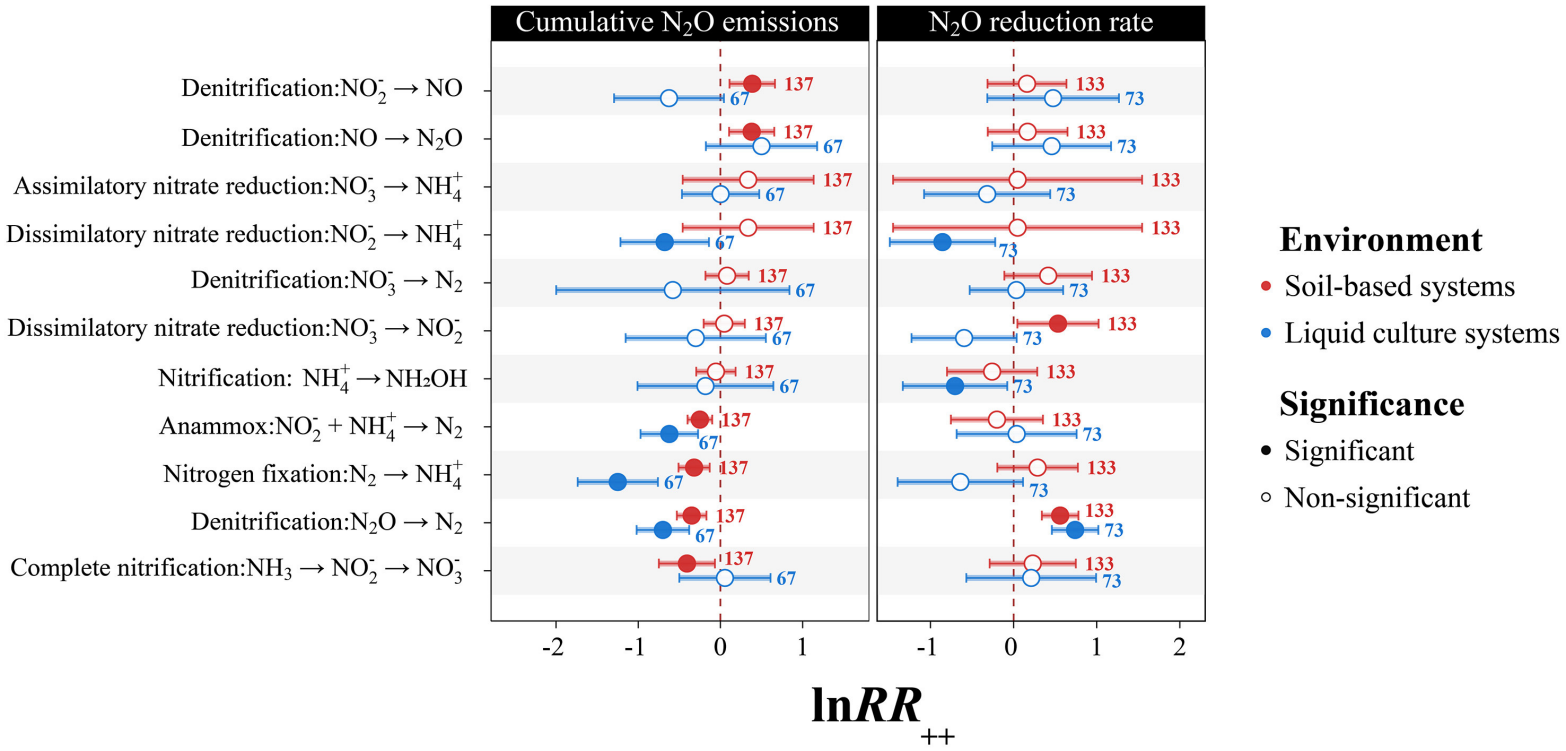
Environment-specific regulatory effects of nitrogen metabolic pathways on N_2_O mitigation. Analysis of regulatory patterns of various nitrogen metabolic pathways across soil-based and liquid culture systems based on functional gene analysis. Red data points represent soil-based systems, and blue data points represent liquid culture systems. The left panel shows the regulatory effects of various nitrogen metabolic pathways on cumulative N_2_O emissions, and the right panel shows the regulatory effects on N_2_O reduction rates. Error bars represent 95% confidence intervals; filled circles indicate significant effects (*p* < 0.05), and empty circles indicate non-significant effects (*p* ≥ 0.05). The *X*-axis represents weighted log response ratio (lnRR_++_), with the dashed line indicating the zero effect line. For cumulative N_2_O emissions, lnRR_++_ < 0 indicates mitigation effects; for N_2_O reduction rates, lnRR_++_ > 0 indicates promotion effects.

Functional gene characterization revealed potential differentiated molecular regulatory patterns of N_2_O mitigation in the two incubation systems (Supplementary Figure S2, Figure 4). The *nosZ* gene exhibited strong N_2_O regulatory capacity in both environments, with clade I *nosZ* showing superior regulatory effects compared to clade II *nosZ* (Supplementary Figure S3). In soil-based systems, strains with nitrifying enzyme genes and nitrate reductase genes both showed significantly enhanced N_2_O mitigation effects compared to strains without these genes (*p* < 0.05). Genomic analysis revealed that genes encoding the two key reaction steps of the anaerobic ammonium oxidation (anammox) pathway (${\text{NH}}_{4}^{+}$ → N_2_H_4_ and N_2_H_4_ → N_2_) were not detected in the genomes of studied genera. However, at the metabolic pathway level, strains with the complete nitrification pathway showed significantly lower N_2_O cumulative emissions compared to strains without this pathway, with an ln*RR*_++_ of −0.42 (*p* < 0.05). Strains carrying genes for denitrification, nitrogen fixation, and anaerobic ammonium oxidation pathways similarly showed reduced N_2_O emissions, with ln*RR*_++_ values of −0.36, −0.34, and −0.26, respectively (*p* < 0.05). For N_2_O reduction rate promotion, dissimilatory nitrate reduction and denitrification pathways performed comparably, with ln*RR*_++_ values of 0.56 and 0.53, respectively (*p* < 0.05).

**Figure 5:**
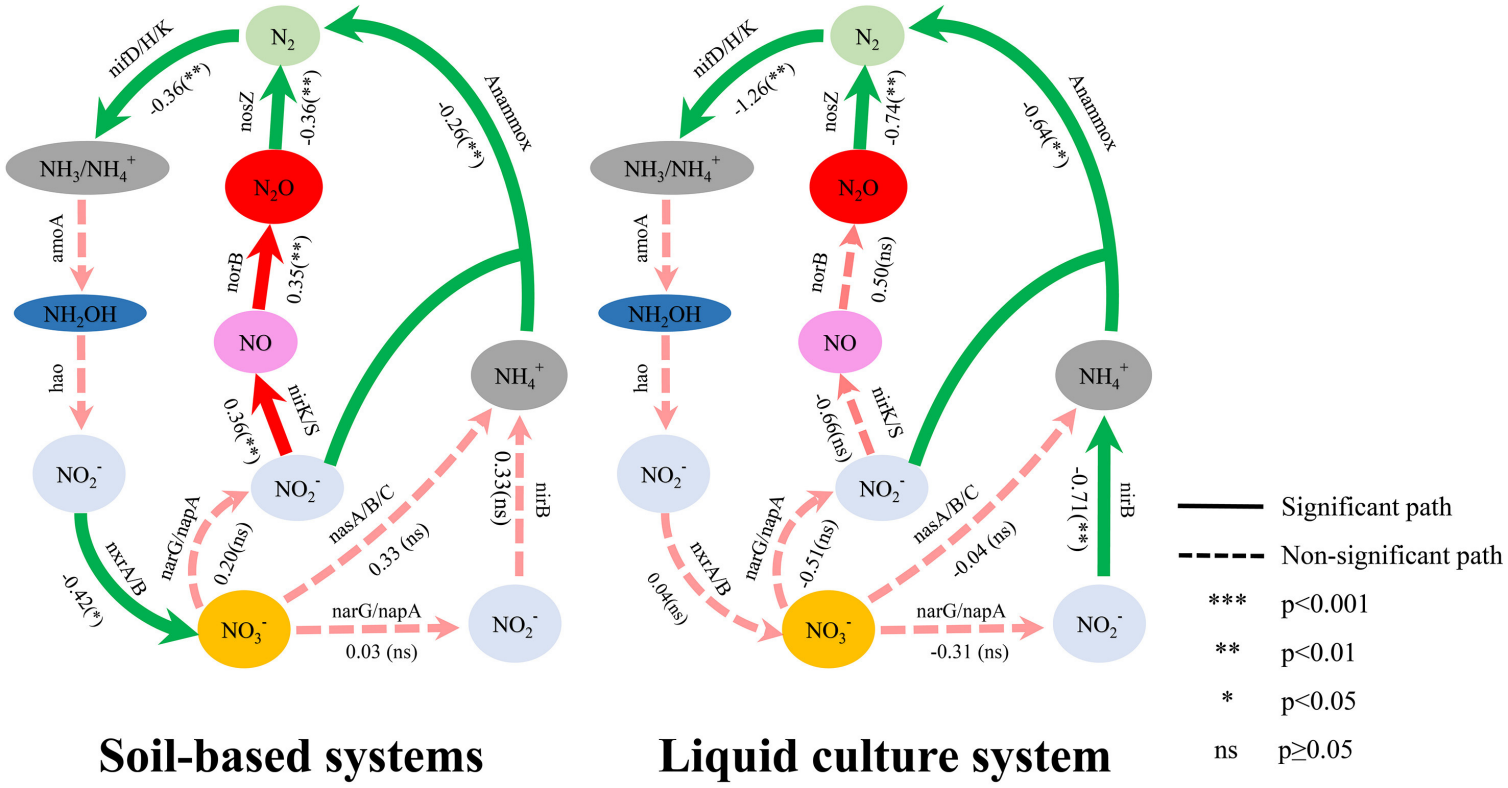
Proposed core metabolic network diagram of N_2_O biological mitigation constructed based on functional gene analysis from this study combined with relevant literature (Xiang, 2024). The diagram centers on N_2_O, showing the regulatory effects of multiple nitrogen cycle pathways on N_2_O production and consumption. Numbers on arrows represent the weighted log response ratio (lnRR_++_) of each pathway on N_2_O cumulative emissions, with gene names indicating the key functional genes controlling each pathway. The left panel shows soil-based systems, and the right panel shows liquid culture systems. Solid arrows represent significant regulatory pathways, and dashed arrows represent non-significant pathways. Significance levels: ***p < 0.001, **p < 0.01, *p < 0.05, ns indicates non-significant (p ≥ 0.05).

Liquid culture systems exhibited different regulatory patterns. Strains with nitrogen fixation genes (*nif* ) showed significantly lower N_2_O emissions compared to strains without these genes (ln*RR*_++_ = −1.25, *p* < 0.05), and at the metabolic pathway level, the mitigation effect of the nitrogen fixation pathway was further enhanced to −1.27 (*p* < 0.05). Strains with the *nirK* gene showed the highest enhancement in N_2_O reduction rates compared to strains without this gene (ln*RR*_++_ = 0.97, *p* < 0.05). The denitrification pathway (N_2_O → N_2_) and anaerobic ammonium oxidation pathway maintained stable mitigation effects in both system types, reflecting the important role of these metabolic pathways in N_2_O regulation. The nitrogen cycle network constructed based on functional gene analysis (Figure 5) showed complex association patterns between key functional genes and N_2_O metabolic processes.

## 4 Discussion

### 4.1 Environment-specific effects and ecological mechanisms of N_2_O mitigation under bacterial inoculation

The pronounced environment-specificity identified through random forest analysis (Figure 1A) reflects fundamental differences in physicochemical constraints and spatial heterogeneity affecting microbial N_2_O metabolism across soil-based and liquid culture systems. This study revealed significant environment-specificity in bacterial inoculation for N_2_O mitigation, with liquid culture systems achieving superior mitigation effects (68.3%) that far exceeded those of soil-based systems (42.9%; Figure 1B). The exceptional mitigation performance in liquid culture systems is primarily attributed to the homogeneous liquid environment ensuring an adequate supply of copper ions required for N_2_O reductase (Zhang et al., 2019), along with stable anaerobic conditions enabling N_2_O reduction processes to proceed across continuous spatial scales, thus avoiding the spatial constraints imposed by oxygen diffusion limitations on denitrification processes in soils (Rohe et al., 2021). Unlike the homogeneous copper-replete anaerobic milieu of liquid cultures, the relatively lower mitigation efficiency in soil-based systems may stem from multiple constraints imposed by environmental factors such as soil pore structure, organic matter distribution, and soil pH on microbial N_2_O metabolism (Hu et al., 2018; Stuchiner et al., 2024; Shaaban et al., 2023). Nevertheless, soil microhabitat heterogeneity provides niche differentiation opportunities for functional microorganisms, with denitrification processes primarily occurring in water-saturated micropores, enabling fine-scale regulation of N_2_O metabolism by soil water-oxygen conditions (Vos et al., 2013; Butterbach-Bahl et al., 2013). These findings suggest that mechanistic insights from liquid culture systems can be applied to flooded soils such as paddy fields, where efficient N_2_O emission mitigation is achievable by maintaining reducing conditions. In contrast, effective mitigation in aerobic soil-based systems such as uplands necessitates overcoming multiple environmental constraints through water management and soil property optimization.

Environment-specificity was also reflected in the temporal differences in microbial community dynamic responses (Figure 2), with soil-based systems requiring medium-term cultivation (10–60 days) to achieve optimal mitigation effects, reflecting the adaptation process whereby inoculated bacteria establish competitive coexistence relationships with native soil microbial communities in complex soil environments (Banerjee and van der Heijden, 2023). The depletion of labile carbon sources during the initial phase may further modulate this temporal pattern, as reduced substrate competition could facilitate the gradual dominance of inoculated functional populations and stabilize long-term N_2_O mitigation (Curtright and Tiemann, 2023; Sennett et al., 2024), collectively suggesting that inoculants should be applied prior to anticipated peak N_2_O emission periods. In contrast, liquid culture systems achieve rapid responses, demonstrating immediate transcriptional activation of functional genes under suitable redox conditions.

Interestingly, this study found that N_2_O mitigation effects under microoxic conditions in soil-based systems were superior to those under anoxic conditions (Figure 2A), providing new insights into understanding the optimal environmental conditions for N_2_O reductase (*nosZ*). Although *nosZ* is considered an anaerobic respiratory enzyme (Zumft, 1997), our results suggest that its functional expression may be optimized under specific microoxic environments. This may be because N_2_O reductase, as an anaerobic respiratory enzyme, is highly sensitive to oxygen (Wang et al., 2023), but oxygen exhibits significantly different regulatory effects on N_2_O production and reduction. While oxygen inhibits N_2_O reductase activity, it more significantly suppresses N_2_O-producing pathways such as incomplete denitrification (Zhu et al., 2013; Ji et al., 2018). In soil environments, except for particularly deep soil layers, most soil layers contain varying degrees of microoxic environments, and this oxygen distribution heterogeneity arises from constraints in gas diffusion and soil aggregate structure (Lacroix et al., 2023), providing an ecological foundation for N_2_O metabolism under microoxic conditions. Under such low oxygen concentration conditions, when oxygen concentration increases, the decline in N_2_O production rate exceeds the decline in consumption rate, resulting in reduced net N_2_O generation in soil and ultimately manifesting as enhanced mitigation effects. On the other hand, in macroscopically microoxic soils, such as within soil aggregates or water film-covered areas (Keiluweit et al., 2017; Wen et al., 2016), numerous anaerobic microenvironments still exist, where bacteria with oxygen-tolerant N_2_O reduction capacity may be enriched (Wang et al., 2023; Takaya et al., 2003), enabling continuous N_2_O reduction under macroscopically microoxic conditions. However, these mechanistic inferences are primarily based on correlations among indicators in the meta-analysis, rather than direct biochemical evidence. Future research needs to verify these hypotheses through direct methods such as oxygen gradient control experiments, real-time monitoring of N_2_O reductase activity, and stable isotope tracing, and elucidate the relative contributions of N_2_O production and consumption processes under different oxygen concentration conditions.

### 4.2 Environmental response of nitrogen cycle functional gene networks and regulatory mechanisms of mitigation pathways

This study systematically explored differences in molecular regulatory networks of N_2_O mitigation across soil-based and liquid culture systems through functional gene characterization and metabolic pathway analysis (Figure 5, Supplementary Figure S2). The N_2_O reduction step, controlled by the *nosZ* gene, exhibited the strongest mitigation effects in both environments, indicating it is the key mechanism for terminal N_2_O elimination, and this validates the core position of the *nosZ* gene as the only known biological N_2_O consumption pathway (Intrator et al., 2024). Gene typing analysis further revealed functional differentiation characteristics (Figure 5, Supplementary Figure S3), with clade I *nosZ* appearing to have stronger N_2_O regulatory efficacy compared to clade II *nosZ*. This may be related to differences in enzymatic kinetic parameters and gene regulatory mechanisms reported in the literature. Clade I *nosZ* is primarily present in traditional denitrifying bacteria and typically possesses higher maximum catalytic rates, while clade II *nosZ*, although active in low N_2_O concentration environments, may have enzymatic kinetic parameters that limit catalytic efficiency when substrate concentrations are high (Sanford et al., 2012; Yoon et al., 2016). Conversely, kinetic studies revealed that clade II organisms exhibit significantly higher substrate affinity for N_2_O compared to clade I organisms, and demonstrate 50-80% higher growth yields per mole of N_2_O reduced, indicating more efficient N_2_O respiration under low substrate concentrations typical of many environments (Yoon et al., 2016). Importantly, the majority of clade I *nosZ* genomes co-occur with nitrite reductase genes, whereas only about half of clade II *nosZ* genomes possess these denitrification genes, suggesting that clade II organisms include a substantial proportion of non-denitrifiers that may consume N_2_O without contributing to its production (Graf et al., 2014; Sanford et al., 2012). Nevertheless, *nosZ* gene functional expression is regulated by multiple environmental conditions including pH and oxygen availability (Hallin et al., 2018). Major differences exist in *nosZ* gene carriage rates among soil denitrifying bacterial communities, with many bacteria possessing only incomplete denitrification capacity where the process terminates at the N_2_O production stage, becoming an important source of net N_2_O emissions (Graf et al., 2014; Orellana et al., 2014). Therefore, the abundance and functional expression of *nosZ* genes should serve as key indicators for denitrification mitigation strategies, providing important molecular foundations for developing precision microbial mitigation technologies and agricultural N_2_O regulation.

Additionally, metabolic pathway analysis further revealed different N_2_O regulatory mechanisms in the two systems (Figure 4). Strains with the complete nitrification pathway showed significantly lower N_2_O cumulative emissions compared to strains without this pathway. This difference may suggest a possible explanatory mechanism that could potentially involve competition between different nitrifying functional groups (Yang et al., 2022). Theoretically, oligotrophic nitrifying bacteria such as comammox *Nitrospira* (Daims et al., 2015; Li et al., 2019) could potentially gain competitive advantages under specific conditions and gradually become the dominant functional group when environmental conditions are favorable, which might inhibit AOB growth and activity, thereby reducing net N_2_O emissions from nitrification processes in soil systems (Liang et al., 2025; Tan et al., 2022). Based on this inference, regulating soil environmental conditions through measures such as precision nitrogen fertilizer management to support the growth of oligotrophic nitrifying bacterial groups could potentially represent an effective indirect mitigation strategy. However, it is important to note that the genomic analysis approach employed in this study cannot definitively distinguish between comammox and traditional two-step nitrification pathways. This explanation is entirely based on functional gene analysis and remains indirect evidence. Therefore, this hypothesis should be regarded as a direction for future research rather than a confirmed mechanism, and urgently requires rigorous validation through direct molecular evidence such as targeted analyses of comammox-specific markers (e.g., amoA gene variants unique to comammox bacteria) (Pjevac et al., 2017), metagenomic sequencing and targeted functional gene quantitative PCR. Meanwhile, nitrate reductase systems further achieve dynamic equilibrium between N_2_O production and consumption through fine-tuned regulation of electron transport chains (Kuypers et al., 2018; Kraft et al., 2014). In contrast, the nitrogen fixation pathway showed prominence in liquid culture systems, reflecting an important indirect regulatory mechanism. Strains carrying *nif* genes showed significantly enhanced N_2_O mitigation effects compared to strains lacking these genes (ln*RR*_++_ = −1.25, Supplementary Figure S2). Biological nitrogen fixation provides nitrogen sources for plants by converting atmospheric N_2_ to ${\text{NH}}_{4}^{+}$, thereby reducing dependence on external nitrogen fertilizers and consequently lowering net N_2_O emissions from the system (Sant'Anna et al., 2018; Minamisawa, 2023). In flooded agricultural systems such as rice paddies, this nitrogen supply strategy based on biological nitrogen fixation not only improves nitrogen use efficiency but also achieves greenhouse gas mitigation benefits at the system level. The anaerobic ammonia oxidation (anammox) pathway directly converts ${\text{NH}}_{4}^{+}$ and ${\text{NO}}_{2}^{-}$ to N_2_ (Kartal et al., 2011), completely bypassing N_2_O as an intermediate product and fundamentally blocking N_2_O generation pathways at the reaction mechanism level, playing an important ecological regulatory role in seasonally anaerobic environments such as rice paddies and wetlands. Although the observed mitigation effects are likely determined by a strain's overall metabolic capacity rather than any single gene or pathway alone, these gene-pathway associations nonetheless provide meaningful mechanistic guidance for environment-specific microbial mitigation strategy selection and targeted N_2_O biological mitigation.

### 4.3 Environmental adaptation and mitigation mechanisms of dominant genera

The differentiated performance of various genera across soil-based and liquid culture systems reflects their unique environmental adaptation strategies and functional gene configurations. *Bradyrhizobium* exhibited exceptional N_2_O reduction capacity in soil-based systems (6.48 μmol·h^−1^·g^−1^, Figure 3C), benefiting not only from its carriage of clade I *nosZ* genes but also from its possession of complete denitrification gene clusters (Sanford et al., 2012; Bedmar et al., 2005) and strong adaptability to complex soil environments. It can cope with osmotic pressure changes caused by wet-dry alternation through synthesis of chemical substances such as trehalose (Ledermann et al., 2021) and possesses the ability to tolerate heavy metal stress and acidic soil environments (Banasiewicz et al., 2025). Meanwhile, it can efficiently colonize the roots of various non-leguminous plants as an endophytic bacterium, occupying rhizosphere microdomains that possess active nutrient cycling and relatively stable moisture (Schneijderberg et al., 2018), thereby maximizing its N_2_O reduction potential. Additionally, this genus participates in rhizosphere nitrogen transformation processes through symbiotic characteristics, utilizing N_2_O reductase to achieve N_2_O reduction (Sánchez and Minamisawa, 2019), further accomplishing mitigation objectives. Based on the synergistic effects of these multiple adaptation mechanisms, *Bradyrhizobium* can fully exert its N_2_O reduction potential under the complex physicochemical conditions of soil-based systems. In contrast, *Azospira* exhibited pronounced environment-specificity, demonstrating significant mitigation advantages in liquid culture systems (mitigation effect value −1.9; reduction rate 3.96 μmol·h^−1^·g^−1^, Figure 3). As a typical representative of the *Rhodocyclaceae* family (Bae et al., 2007), it is a facultative anaerobic bacterium harboring complete denitrification and nitrogen fixation gene clusters (Suenaga et al., 2018a) and carrying clade II *nosZ* genes (Suenaga et al., 2018b). Its physiological and metabolic characteristics are highly compatible with the anaerobic nature, high substrate diffusivity, and neutral pH of liquid culture environments (Zhou et al., 2022). Flagellar motility enables effective migration in liquid environments and efficient utilization of electron acceptors such as nitrate for denitrification metabolism (Bae et al., 2007), thereby achieving efficient N_2_O reduction in this environment. Therefore, the excellent N_2_O mitigation capacity of *Azospira* in liquid systems highlights its considerable potential as a candidate bacterial group for greenhouse gas mitigation in flooded agricultural environments such as paddy fields and wetlands. However, while flooded paddy environments share similar anaerobic conditions with the liquid culture systems favorable for *Azospira*'s denitrification activity, the field performance and practical applicability of this genus cannot be directly extrapolated from liquid culture results (Herrmann and Lesueur, 2013). Future research must prioritize *in situ* field trials to systematically evaluate its establishment success, competitive fitness against indigenous microbiota (Mawarda et al., 2020), and actual mitigation efficacy under natural field soil conditions, thereby bridging the gap between laboratory-demonstrated potential and practical agricultural applications.

This study revealed that different genera exhibit environment-specific N_2_O mitigation effects through differentiated physiological mechanisms. *Gemmatimonas* possesses a unique oxygen-dependent N_2_O reduction mechanism, enabling flexible adjustment of N_2_O metabolic activity according to environmental oxygen conditions (Oshiki et al., 2022), thereby maintaining mitigation capacity across different environmental systems. Conversely, *Bacillus* showed performance contrary to the overall trend, exhibiting higher N_2_O reduction activity in soil-based systems (2 μmol·h^−1^·g^−1^, Figure 3C), which may stem from its unique sporulation physiological mechanism, where spore germination delays *nosZ* gene expression, limiting the expression of mitigation potential in nutrient-rich liquid culture systems (Bressuire-Isoard et al., 2018).

Based on the environment-specific performance among genera, this study revealed the crucial role of differentiated strain selection in N_2_O mitigation. Precise matching between environmental conditions and strain functions is a prerequisite for achieving efficient mitigation, and this finding provides a theoretical foundation for developing environment-adaptive microbial mitigation technologies, contributing to further optimization of agricultural N_2_O mitigation strategies.

## 5 Conclusion

This study systematically evaluated the N_2_O mitigation potential of bacterial inoculation across diverse agricultural environments through meta-analysis integrating 257 data points from 34 independent studies. Our findings demonstrate that among all environmental variables examined, environmental system serves as the primary driver governing mitigation outcomes, a finding that subsequently guided the focus toward two distinct systems characterized by contrasting mitigation performance. The results reveal that liquid culture systems achieve significantly superior mitigation effects (68.3%) compared to soil-based systems (42.9%), demonstrating pronounced environment-specificity. The *nosZ* gene-mediated denitrification pathway appears to represent the common core mechanism for mitigation in both systems based on functional gene analysis, while dissimilatory nitrate reduction pathway and nitrogen fixation pathway play key regulatory roles in soil-based and liquid culture systems, respectively. Phylogenetic analysis indicates that *Bradyrhizobium*, with its soil adaptability and complete functional gene configuration, represents the preferred strain for soil-based systems, while *Azospira* shows promise as a candidate bacterial group for liquid-dominated environments such as paddy fields and wetlands. The microbial N_2_O mitigation environmental regulatory network revealed by this study provides theoretical foundations for differentiated strain selection and N_2_O mitigation strategy optimization, offering important guidance for mitigating agricultural greenhouse gas emissions.

This study has certain methodological limitations. The functional gene analysis was conducted at the genomic level, representing genetic potential rather than actual gene expression or enzymatic activity in soil environments. Gene presence does not guarantee functional expression, as activity depends on environmental factors (oxygen, pH, substrate availability) and microbial community interactions. Additionally, for strains lacking genome sequences, representative genomes from the same genus were used as proxies, which may introduce uncertainty given intra-genus genetic diversity. Future research should integrate metagenomics to assess community-level functional gene profiles, metatranscriptomics to quantify actual gene expression under field conditions, and long-term field trials to validate mitigation stability and ecological sustainability. Translating laboratory-based strain recommendations to field applications will further require dedicated attention to colonization efficiency and competitive persistence of inoculated strains within indigenous soil microbial communities. Collectively, addressing these limitations will be essential for advancing environment-specific microbial N_2_O mitigation technologies from laboratory-demonstrated potential toward practical agricultural implementation.

## Data Availability

The original contributions presented in the study are included in the article/Supplementary material, further inquiries can be directed to the corresponding authors.
