# Supplementary material for: Bacterial N2O mitigation potential in soil-based systems and liquid cultures: a comprehensive meta-analysis
Source: Front Microbiol. 2026 Apr 8;17:1803828. doi: 10.3389/fmicb.2026.1803828 (PMC13099815; doi:10.3389/fmicb.2026.1803828)
Supplement: Supplementary file 1 [file Data_Sheet_1.docx]

**Bacterial N₂O mitigation potential in soil-based systems and liquid cultures:**

**A comprehensive meta-analysis**

Shengsen Zhou ^a^, Ruixuan Zhu ^a^, Yongfeng Sun ^a^, Dingjiao Peng ^a^, Fumin Wei ^a^, Xiaomai Yuan ^a^, Beilei Wei ^a^, Dan Lu ^b^, Weiwei Li ^c *^, Yu Jiang ^c *^, Ziting Wang ^a *^

^a^ State Key Lab for Conservation and Utilization of Subtropical Agri-Biological Resources, Guangxi Key Lab for Sugarcane Biology, College of Agriculture, Guangxi University, Nanning, 530004, China

^b^ College of Agriculture, Guangxi University, Nanning, 530004, China

^c^ Collaborative Innovation Center for Modern Crop Production / Key Laboratory of Crop Physiology and Ecology in Southern China / State Key Laboratory of Crop Genetics & Germplasm Enhancement and Utilization, Nanjing Agricultural University, Nanjing, 210095, China

^*^ Corresponding author. Email: zitingwang@gxu.edu.cn (Z.T. Wang)

**Table S1** Results of publication bias about datasets in this study. “N” is the number of observations.

| **Factors** | **N** | **Rosenthal Fail-safe number** | **P** | **5n + 10** | **Publication bias** |
| --- | --- | --- | --- | --- | --- |
| Cumulative N_2_O emissions | 212 | 6655 | <0.0001 | 1070 | No |
| N_2_O reduction rate | 214 | 15973 | <0.0001 | 1080 | No |
| NH+ 4 | 41 | 0 | 0.4374 | 215 | Not applicable |
| NO- 3 | 43 | 0 | 0.2692 | 225 | Not applicable |
| PNR | 4 | 0 | 0.1934 | 30 | Not applicable |
| PDR | 42 | 141 | 0.0003 | 220 | Potential concern |
| nirK | 60 | 0 | 0.9215 | 310 | Not applicable |
| nirS | 59 | 0 | 0.3818 | 305 | Not applicable |
| nosZ | 62 | 0 | 0.9761 | 320 | Not applicable |
| nosZ/(nirK+nirS) | 59 | 97 | 0.0116 | 305 | Potential concern |

**Table S2**. Heterogeneity test results for physicochemical factors affecting N₂O emissions and reduction rates across soil-based and liquid culture systems. Physicochemical factors include pH regimes, oxygen status, cultivation time, and soil nutrient properties (TC, NO₃⁻-N, NH₄⁺-N, AP, AK); detailed classifications are provided in Methods section 2.4.2. Analyses were conducted separately for soil-based and liquid culture systems. N: number of observations; QM: between-group heterogeneity statistic; I²: heterogeneity index (%); p_label: significance level (***p < 0.001, **p < 0.01, *p < 0.05, ns: non-significant).

| **Environment** | **Response indicators** | **subgroups** | **N** | **QM** | **I_2_(%)** | **p_label** |
| --- | --- | --- | --- | --- | --- | --- |
| **soil-based systems** | N_2_O reduction rate | pH | 160 | 27.49 | 99.91 | *** |
|  |  | TN | 98 | 6.07 | 99.82 | * |
|  |  | TC | 119 | 9.37 | 99.68 | ** |
|  |  | NH_4_^+^ | 109 | 15.54 | 99.82 | *** |
|  |  | NO_3_^-^ | 114 | 0.010 | 99.85 | ns |
|  |  | AP | 79 | 0.337 | 99.76 | ns |
|  |  | AK | 77 | 6.19 | 99.74 | * |
|  |  | cultivation time | 141 | 14.76 | 99.92 | *** |
|  |  | Oxygen status | 160 | 148.54 | 99.92 | *** |
|  | Cumulative N_2_O emissions | pH | 146 | 13.25 | 99.63 | *** |
|  |  | TN | 114 | 0.79 | 99.67 | ns |
|  |  | TC | 119 | 13.62 | 97.49 | *** |
|  |  | NH_4_^+^ | 127 | 23.52 | 99.58 | *** |
|  |  | NO_3_^-^ | 132 | 7.69 | 99.65 | ** |
|  |  | AP | 83 | 5.09 | 96.58 | * |
|  |  | AK | 81 | 1.77 | 96.57 | ns |
|  |  | cultivation time | 145 | 0.40 | 99.67 | ns |
|  |  | Oxygen status | 146 | 85.17 | 99.64 | *** |
| **liquid culture systems** | N_2_O reduction rate | pH | 76 | 0.08 | 99.93 | ns |
|  |  | cultivation time | 73 | 0.01 | 99.93 | ns |
|  |  | Oxygen status | 76 | 165.26 | 99.93 | *** |
|  | Cumulative N_2_O emissions | pH | 67 | 7.59 | 99.86 | ** |
|  |  | cultivation time | 67 | 1.47 | 99.87 | ns |
|  |  | Oxygen status | 67 | 94.80 | 99.85 | *** |


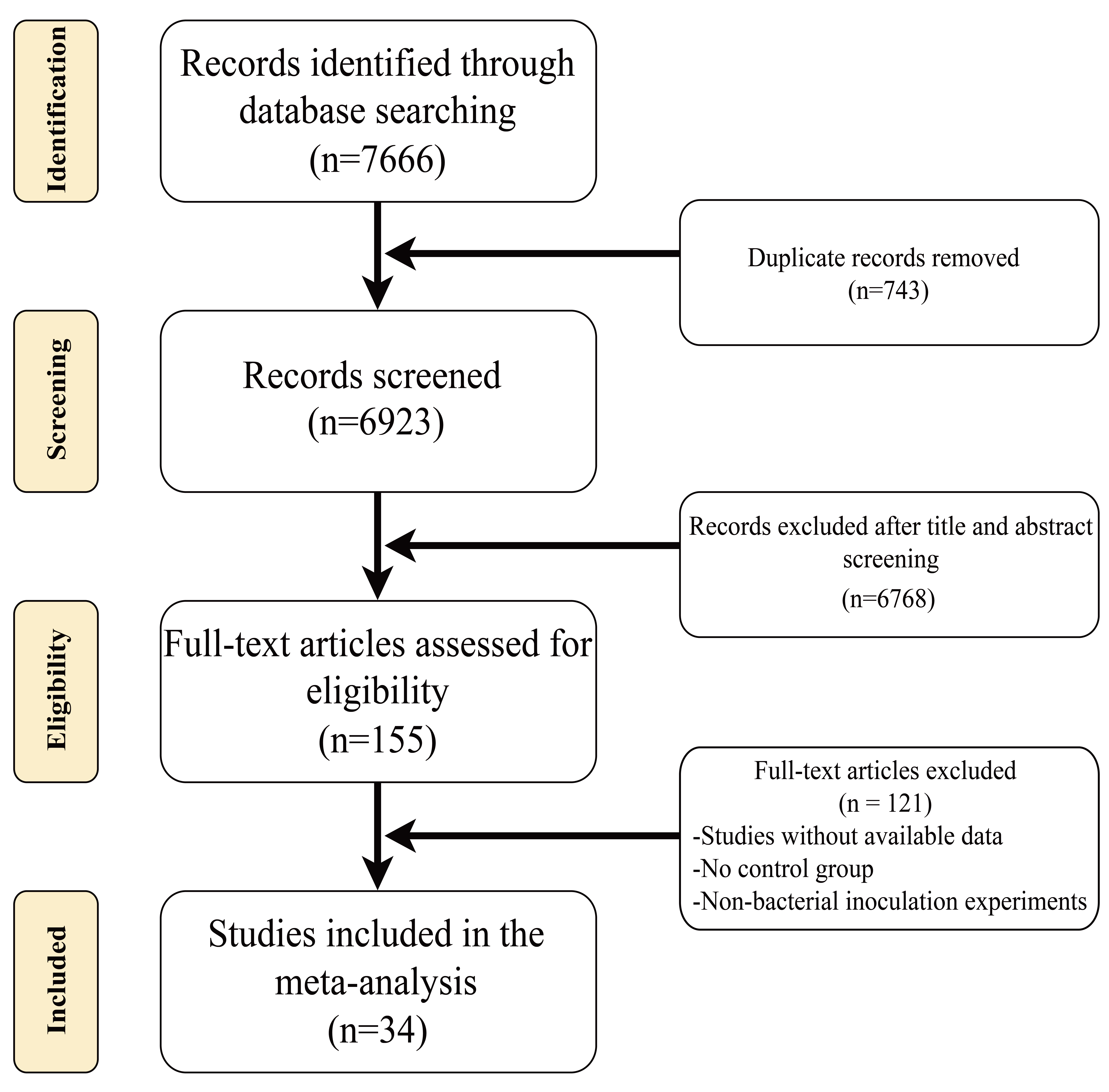


**Fig. S1**. PRISMA flow diagram of the literature search and selection process.





**Fig. S2**. Differential regulatory effects of nitrogen cycling functional genes on N₂O metabolism under different soil conditions. The left panel shows the effects of functional genes on cumulative N₂O emissions, while the right panel shows their effects on N₂O reduction rates. Red represents unsaturated soil systems (upland agricultural environments), and blue represents water-saturated systems (paddy field/wetland environments). The x-axis represents weighted log response ratio (ln*RR_++_*), with the dashed line (ln*RR_++_* = 0) indicating the null effect. For cumulative N₂O emissions, ln*RR_++_* < 0 indicates emission reduction effects; for N₂O reduction rates, ln*RR_++_* > 0 indicates promotion of reduction. Filled circles indicate significant effects (p<0.05), while empty circles indicate non-significant effects (p≥0.05). Error bars represent 95% confidence intervals.


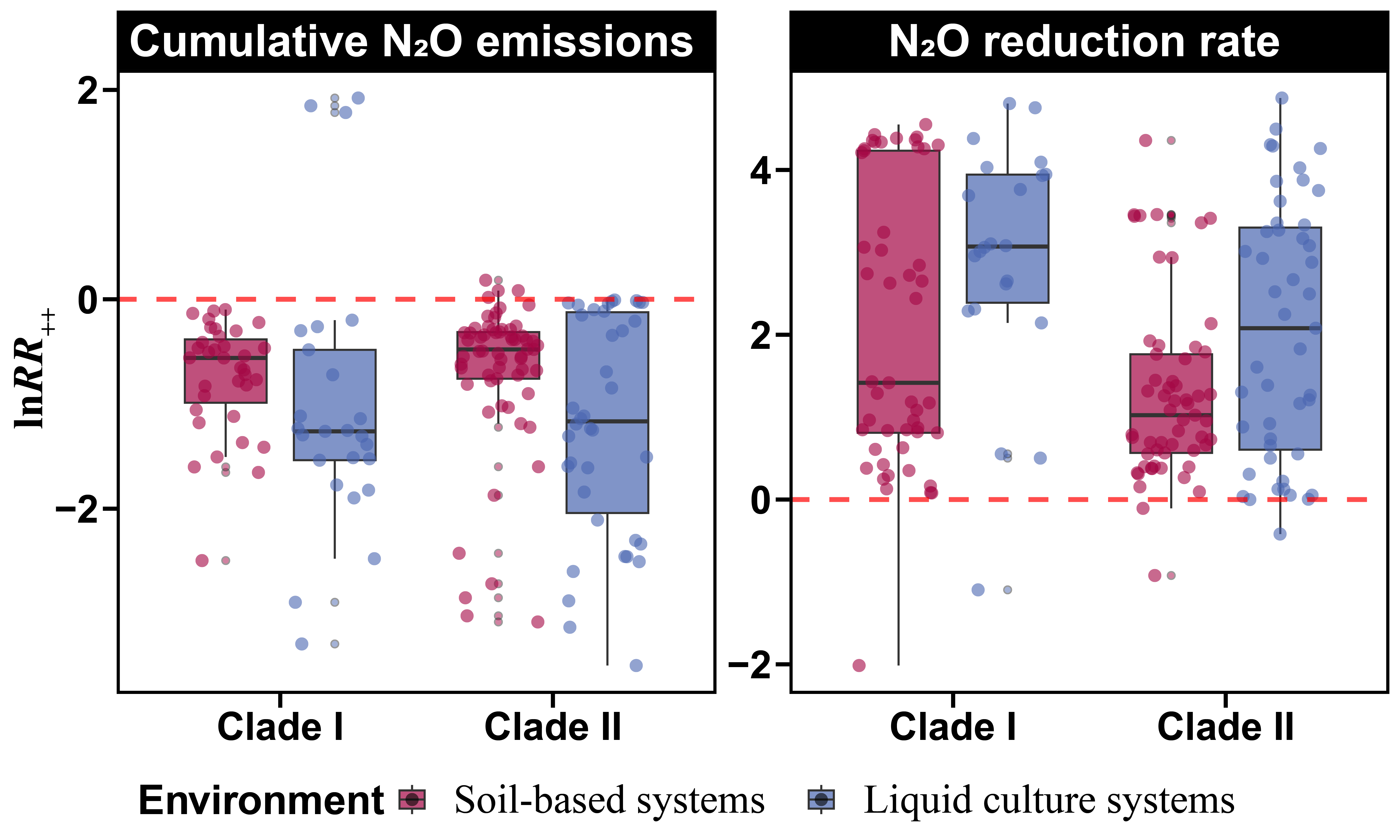


**Fig. S3**. Comparison of N₂O regulatory effects between *nosZ* gene clades across different soil environments. Left panel shows weighted mean log response ratios (ln*RR_++_*) for cumulative N₂O emissions, and right panel shows ln*RR_++_* for N₂O reduction rates. Clade I and Clade II represent the two major phylogenetic clades of *nosZ* genes. Red indicates unsaturated soil systems, and blue indicates water-saturated systems. Box plots display median, interquartile ranges, and data distribution, with individual data points shown as scatter plots. Red dashed line represents the null effect line (ln*RR_++_* = 0). For cumulative N₂O emissions, ln*RR_++_* < 0 indicates mitigation effects; for N₂O reduction rates, ln*RR*_++_ > 0 indicates promotion effects.

**Text S1** List of all articles included in this meta-analysis. The blue fonts represent the articles written in Chinese. The articles that were uncited in the main text were marked with terminal asterisks in parentheses.

Behrendt, U., Spanner, T., Augustin, J., Ulrich, A., Kitz, F., Glatzel, S., et al. (2022). Consumption of N_2_O by *Flavobacterium azooxidireducens* sp. nov. Isolated from Decomposing Leaf Litter of *Phragmites australis* (Cav.). *Microorganisms* 10:2304. doi: 10.3390/microorganisms10112304 (*)

Chee-Sanford, J., Tian, D., and Sanford, R. (2019). Consumption of N_2_O and other N-cycle intermediates by *Gemmatimonas aurantiaca* strain T-27. *Microbiology* 165, 1345–1354. doi: 10.1099/mic.0.000847 (*)

Domeignoz-Horta, L. A., Putz, M., Spor, A., Bru, D., Breuil, M. C., Hallin, S., et al. (2016). Non-denitrifying nitrous oxide-reducing bacteria - an effective N₂O sink in soil. *Soil Biol. Biochem.* 103, 376–379. doi: 10.1016/j.soilbio.2016.09.010 (*)

Fukushi, M., Mino, S., Tanaka, H., Kouzuma, A., Nakagawa, S., Chiba, Y., et al. (2020). Biogeochemical implications of N_2_O-reducing thermophilic Campylobacteria in deep-sea vent fields, and the description of *Nitratiruptor labii* sp. nov. *iScience* 23:101462. doi: 10.1016/j.isci.2020.101462 (*)

Gao, N., Shen, W., Camargo, E., Shiratori, Y., Hattori, S., Nishizawa, T., et al. (2017). Nitrous oxide (N_2_O)-reducing denitrifier-inoculated organic fertilizer mitigates N_2_O emissions from agricultural soils. *Biol. Fertil. Soils* 53, 885–898. doi: 10.1007/s00374-017-1231-z (*)

Gao, N., Zhang, H., Hu, C., Wang, Y., Cai, C., Liu, H., et al. (2024). Inoculation with *Stutzerimonas stutzeri* strains decreases N₂O emissions from vegetable soil by altering microbial community composition and diversity. *Microbiol. Spectr.* 12:e00186-24. doi: 10.1128/spectrum.00186-24 (*)

Han, Z., Leng, Y., Sun, Z., Bai, J., Zheng, C., Wang, H., et al. (2024). Substitution of organic and bio-organic fertilizers for mineral fertilizers to suppress nitrous oxide emissions from intensive vegetable fields. *J. Environ. Manage.* 349:119390. doi: 10.1016/j.jenvman.2023.119390 (*)

Hiis, E. G., Vick, S. H. W., Molstad, L., Røsdal, K., Jonassen, K. R., Winiwarter, W., et al. (2024). Unlocking bacterial potential to reduce farmland N_2_O emissions. *Nature* 630, 421–428. doi: 10.1038/s41586-024-07464-3

Huang, M., Zhang, Y., Wu, J., Liu, J., Wang, Y., Liang, Y., et al. (2023). *Bacillus velezensis* SQR9 inhibition to fungal denitrification responsible for decreased N₂O emissions from acidic soils. *Sci. Total Environ.* 885:163789. doi: 10.1016/j.scitotenv.2023.163789 (*)

Hu, C., Fang, Y., Zhang, H. H., Shen, Z. Y., Zhang, M. M., Liu, R., et al. (2024). Effects of two plant growth promoting rhizobacterium strains on nitrous oxide emissions in maize soil. *Soil Fertil. Sci. China* 11, 57–69. doi: 10.11838/sfsc.1673-6257.24073 (*)

Hu, L., Wang, X., Chen, C., Lin, Y., Liang, F., Lin, K., et al. (2022). *NosZ* gene cloning, reduction performance and structure of *Pseudomonas citronellolis* WXP-4 nitrous oxide reductase. *RSC Adv.* 12, 2549–2557. doi: 10.1039/d1ra09008a (*)

Itakura, M., Uchida, Y., Akiyama, H., Hoshino, Y. T., Shimomura, Y., Morimoto, S., et al. (2013). Mitigation of nitrous oxide emissions from soils by *Bradyrhizobium japonicum* inoculation. *Nat. Clim. Change* 3, 208–212. doi: 10.1038/nclimate1734 (*)

Jiang, M., Zheng, X., and Chen, Y. (2020). Enhancement of denitrification performance with reduction of nitrite accumulation and N_2_O emission by *Shewanella oneidensis* MR-1 in microbial denitrifying process. *Water Res.* 169:115242. doi: 10.1016/j.watres.2019.115242 (*)

Ji, C., Wang, J., Sun, Y., Wang, P., Shao, M., Wang, H., et al. (2024). Wheat straw and microbial inoculants have an additive effect on N₂O emissions by changing microbial functional groups. *Eur. J. Soil Sci.* 75:e13494. doi: 10.1111/ejss.13494 (*)

Kim, H., Park, D., and Yoon, S. (2017). pH control enables simultaneous enhancement of nitrogen retention and N_2_O reduction in *Shewanella loihica* Strain PV-4. *Front. Microbiol.* 8:1820. doi: 10.3389/fmicb.2017.01820 (*)

Kim, J. Y., and Cho, K. S. (2022). Inoculation effect of *Pseudomonas* sp. TF716 on N_2_O emissions during rhizoremediation of diesel-contaminated soil. *Sci. Rep.* 12:13018. doi: 10.1038/s41598-022-17356-z (*)

Liu, C. M., Sheng, R., Liu, Y., An, Y., Chen, Y., Chai, R., et al. (2018). Capability of N_2_O reduction of a facultative N_2_O reducer. *Acta Microbiol. Sin.* 58, 1431–1438. doi: 10.13343/j.cnki.wsxb.20170482 (*)

Li, Q., Wang, Z. Z., Zhang, H. H., Li, L. M., Gao, N., Wu, G. P., et al. (2025). Effects of four plant growth promoting rhizobacteria on soil N_2_O emissions from tomato facilities. *J. Nanjing Univ. Inf. Sci. Technol.* 17, 443–454. doi: 10.13878/j.cnki.jnuist.20240430002 (*)

Mauffrey, F., Cucaita, A., Constant, P., and Filion, M. (2017). Denitrifying metabolism of the methylotrophic marine bacterium *Methylophaga nitratireducenticrescens* strain JAM1*. PeerJ* 5:e4098. doi: 10.7717/peerj.4098 (*)

Oba, K., Yasuda, S., and Terada, A. (2024). Complete genome sequence of *Afipia carboxidovorans* strain SH125, a non-denitrifying nitrous oxide-reducing bacterium isolated from anammox biomass. *Microbiol. Resour. Announc.* 13:e01279-23. doi: 10.1128/mra.01279-23 (*)

Park, D., Kim, H., and Yoon, S. (2017). Nitrous oxide reduction by an obligate aerobic bacterium, *Gemmatimonas aurantiaca* strain T-27. *Appl. Environ. Microbiol.* 83:e00502-17. doi: 10.1128/AEM.00502-17 (*)

Park, H.-J., Kwon, J. H., Yun, J., and Cho, K.-S. (2020). Characterization of nitrous oxide reduction by *Azospira* sp. HJ23 isolated from advanced wastewater treatment sludge. *J. Environ. Sci. Health Part A* 55, 1459–1467. doi: 10.1080/10934529.2020.1812321 (*)

Philippot, L., Andert, J., Jones, C. M., Bru, D., and Hallin, S. (2011). Importance of denitrifiers lacking the genes encoding the nitrous oxide reductase for N₂O emissions from soil. *Glob. Change Biol.* 17, 1497–1504. doi: 10.1111/j.1365-2486.2010.02334.x (*)

Semedo, M., Wittorf, L., Hallin, S., Veríssimo, A., and Song, B. (2020). Differential expression of clade I and II N_2_O reductase genes in denitrifying *Thauera linaloolentis* 47LolT under different nitrogen conditions. *FEMS Microbiol. Lett.* 367:fnaa205. doi: 10.1093/femsle/fnaa205 (*)

Suenaga, T., Aoyagi, R., Sakamoto, N., Riya, S., Terada, A., and Hosomi, M. (2018). Immobilization of *Azospira* sp. strain I13 by gel entrapment for mitigation of N₂O from biological wastewater treatment plants: biokinetic characterization and modeling. *J. Biosci. Bioeng.* 126, 213–219. doi: 10.1016/j.jbiosc.2018.02.014 (*)

Tao, R., Wakelin, S. A., Liang, Y., Hu, B., and Chu, G. (2018). Nitrous oxide emission and denitrifier communities in drip-irrigated calcareous soil as affected by chemical and organic fertilizers. *Sci. Total Environ.* 612, 739–749. doi: 10.1016/j.scitotenv.2017.08.258 (*)

Tian, W., Cheng, Z. L., Zhou, Q., Ye, Y. S., Liu, S. W., Wang, J. Y., et al. (2024). Potential and mechanisms of bio-organic fertilizer in reducing N_2_O emission from vegetable soil. *J. Agric. Resour. Environ.* 42, 369–379. doi: 10.13254/j.jare.2024.0008 (*)

Wang, F., Xu, S. J., Ma, S. L., Sun, H., Yin, Y. X., Li, C. Y., et al. (2015). Effect of *Bacillus amyloliquefaciens* Biofertilizer on *Brassica juncea* var. *multiceps* Growth and N_2_O Emission from Soil. *Chin. Agric. Sci. Bull.* 31, 229–235. doi: 10.11924/J.issn.1000-6850.2014-2479 (*)

Wang, Y., Deng, M., Li, B., Li, Y., Chen, D., Sun, L., et al. (2023). High nitrous oxide (N₂O) greenhouse gas reduction potential of *Pseudomonas* sp. YR02 under aerobic condition. *Bioresour. Technol.* 378:128994. doi: 10.1016/j.biortech.2023.128994 (*)

Wasai-Hara, S., Itakura, M., Siqueira, A. F., Murase, M., Nagasaki, S., Matsuda, S., et al. (2023). *Bradyrhizobium ottawaense* efficiently reduces nitrous oxide through high nosZ gene expression. *Sci. Rep.* 13:18862. doi: 10.1038/s41598-023-46019-w (*)

Wu, S., Zhuang, G., Bai, Z., Cen, W., Peng, X., and Zhuang, X. (2018). Mitigation of nitrous oxide emissions from acidic soils by *Bacillus amyloliquefaciens*, a plant growth-promoting bacterium. Glob. *Change Biol.* 24, 2352–2365. doi: 10.1111/gcb.14025 (*)

Zhou, J., Deng, W., Wu, J., Liu, L., Zhang, L., Yang, L., et al. (2024). Respiration and growth of *Paracoccus denitrificans* R-1 with nitrous oxide as an electron acceptor. *Microbiol. Spectr.* 12:e03811-23. doi: 10.1128/spectrum.03811-23 (*)

Zhou, S., Zeng, X., Xu, Z., Xu, J., Jiang, Y., Xiao, T., et al. (2020). *Paenibacillus polymyxa* biofertilizer application in a tea plantation reduces soil N_2_O by changing denitrifier communities. Can. *J. Microbiol.* 66, 214–227. doi: 10.1139/cjm-2019-0511 (*)

Zhu, J. H., Xiong, R. N., Yang, S. Q., He, T., Tian, Y., Li, J. Y., et al. (2024). Effects of inoculation with N_2_O-reducing bacteria YSQ030 on soil N_2_O emission and key functional genes involved in nitrogen cycling in reclaimed soil. *J. Nanjing Univ. Inf. Sci. Technol.* 16, 416–427. doi: 10.13878/j.cnki.jnuist.20230312001 (*)
